# An Electrochemical Sensor Based on Chalcogenide Molybdenum Disulfide-Gold-Silver Nanocomposite for Detection of Hydrogen Peroxide Released by Cancer Cells

**DOI:** 10.3390/s20236817

**Published:** 2020-11-28

**Authors:** Jinchun Hu, Congcong Zhang, Xue Li, Xin Du

**Affiliations:** Shandong Provincial Key Laboratory of Animal Resistance Biology, Key Laboratory of Food Nutrition and Safety, College of Life Sciences, Shandong Normal University, Jinan 250014, China; hujinchun_369@163.com (J.H.); zhangcc1026@163.com (C.Z.); lx1213940737@163.com (X.L.)

**Keywords:** hydrogen peroxide, tumor cells, molybdenum disulfide–gold–silver, electrochemical sensor, detection

## Abstract

Hydrogen peroxide (H_2_O_2_) as a crucial signal molecule plays a vital part in the growth and development of various cells under normal physiological conditions. The development of H_2_O_2_ sensors has received great research interest because of the importance of H_2_O_2_ in biological systems and its practical applications in other fields. In this study, a H_2_O_2_ electrochemical sensor was constructed based on chalcogenide molybdenum disulfide–gold–silver nanocomposite (MoS_2_-Au-Ag). Transmission electron microscopy (TEM), X-ray photoelectron spectroscopy (XPS) and energy dispersive spectroscopy (EDS) were utilized to characterize the nanocomposites, and the electrochemical performances of the obtained sensor were assessed by two electrochemical detection methods: cyclic voltammetry and chronoamperometry. The results showed that the MoS_2_-Au-Ag-modified glassy carbon electrode (GCE) has higher sensitivity (405.24 µA mM^−1^ cm^−2^), wider linear detection range (0.05–20 mM) and satisfactory repeatability and stability. Moreover, the prepared sensor was able to detect the H_2_O_2_ discharge from living tumor cells. Therefore, this study offers a platform for the early diagnosis of cancer and other applications in the fields of biology and biomedicine.

## 1. Introduction

Cells can produce all kinds of reactive oxygen species in metabolic process, and H_2_O_2_ is one of the most momentous representatives. H_2_O_2_ plays a vital role in numerous processes such as cellular signal transduction, apoptosis and many other physiological processes [1]; however, excess H_2_O_2_ is toxic to humans, which leads to various diseases, such as cancer, cardiovascular disease and neurodegenerative diseases [2]. Research has proved that cancer cells will release excessive H_2_O_2_ to achieve the abnormal growth of tumors. Therefore, the accurate and fast determination of H_2_O_2_ is significant in the cellular environment and diagnosing cancer in its early stages. Additionally, H_2_O_2_ is widely used as an oxidizer, disinfectant or bleach in the food, environmental analysis, chemical and pharmaceutical industries [3,4].

The traditional methods for the detection of H_2_O_2_ include spectrophotometry [5], chemiluminescence [6,7], fluorescence [8,9] and high performance liquid chromatography [10]. However, these methods also have some limitations, such as expensive equipment and cumbersome procedures. Electrochemical detection is a detective method which has the advantages of high sensitivity, low cost, high efficiency and good portability [11]. In recent years, the development of nanomaterials has provided many new opportunities for the construction of hydrogen peroxide sensors. Nanomaterials have the unique properties of a strong adsorption capacity, large specific surface area and high catalytic efficiency [12,13]. In the field of electrochemical sensors, the application of nanomaterials not only improves the stability and sensitivity of electrodes, but also improves the detection performance of sensors [13].

MoS_2_ has attracted extensive attention in the studies of lithium batteries, catalysts and cancer treatment owing to its advantages of good stability, high catalytic efficiency and easy functionalization [14,15,16]. The structure of single-layer MoS_2_ is composed of two layers of sulfur sandwiched by a layer of molybdenum. The atoms are covalently bonded to each other, which shows as a sandwich structure [17]. At present, the sensors using molybdenum disulfide as the experimental material are involved in the detection of glucose, hydrogen peroxide, nucleic acid, protein and other substances which showed a good sensitivity and detection performance. Au, Ag and other precious metal nanomaterials not only have the characteristics of general nanometer materials, but also have excellent electrical conductivity, a unique catalytic activity and are non-toxic, which makes them have unique advantages and extensive applications in the field of sensors [18,19].

Herein, we developed a sensitive sensor for the detection of H_2_O_2_ discharge from tumor cells by using a MoS_2_-Au-Ag nanocomposite to accomplish high conductivity and increase the surface area of the electrode, due to the fact that H_2_O_2_ is a typical tumor biomarker and the developed MoS_2_-Au-Ag electrode exhibits an excellent enzyme-mimic electrocatalytic activity in the oxidation of H_2_O_2_. We chose cyclic voltammetry (CV) to evaluate the properties of our fabricated sensor for the detection of H_2_O_2_, which is a very sensitive electrochemical detection tool used to evaluate the performance of an electrochemical sensor [19]. Our results indicated that this sensor significantly improved the detection signal and consequently enhanced the low detection limit with a wide detection range, and high selectivity and sensitivity. This study demonstrates a functional, non-enzymatic electrochemical sensor based on MoS_2_-Au-Ag nanocomposite for the detection of H_2_O_2_ released by normal and tumor cells.

## 2. Experimental

### 2.1. Chemicals and Reagents

MoS_2_, phthalic diglycol diacrylate (PDDA), K_3_[Fe(CN)_6_], phorbol 12-myristate 13-acetate (PMA), chloroauric acid (H[AuCl_4_]) and AgNO_3_ were obtained from Sigma (USA). H_2_O_2_ was purchased from Shanghai Wokai Biotechnology Co., Ltd. (China). C_2_H_5_OH was obtained from Tianjin Chemical Reagent Sixth Factory (China). KCl was purchased from Shanghai Hutan Laboratory Equipment Co., Ltd. (China). Phosphate buffer solution (PBS) was obtained from Biological Industries. 3-(4,5-dimethylthiazol-2-yl)-2,5-diphenyltetrazolium bromide (MTT) was obtained from Beijing Solarbio Science & Technology Co., Ltd. (China). The RPE-1 human retinal pigment epithelial cell line and the MCF-7 human breast cancer cell line were purchased from the cell bank of the Chinese Academy of Sciences (Shanghai, China). For the configuration of all aqueous solutions and to clean all materials we used twofold distilled water.

### 2.2. Electrochemical Measurements

The electrochemical detection used a standard three-electrode electrochemical testing device with a glassy carbon electrode modified by nanomaterials as the working electrode, and a silver/silver chloride (Ag/AgCl) and platinum wire were used as the reference electrode and counter electrode, respectively. The electrochemical test was conducted in PBS or cell samples, which were deoxygenated with N_2_ and stirred with a magnetic agitator at a speed of 300 rpm to maintain a stable and uniform H_2_O_2_ concentration in the solution.

### 2.3. Dispersion of MoS_2_

The dispersing agent PDDA was used to disperse the MoS_2_ into a uniform and stable dispersion by taking 3 mg and 7.5 mg MoS_2_ into a 1.5 mL centrifuge tube and then adding 1% or 0.5% PDDA at a constant volume to 1.5 mL. Next, we got a MoS_2_ solid precipitate via sonication for 2 h and centrifuged it at 15,000 rpm for 10 min. Subsequently, four different concentrations of dispersion were obtained by discarding the supernatant and adding water at a constant volume to 1.5 mL, including 2 mg mL^−1^ and 5 mg mL^−1^ MoS_2_ dispersions with 0.5% and 1% PDDA.

### 2.4. Preparation of MoS_2_-Au-Ag/GCE

**Scheme 1:**
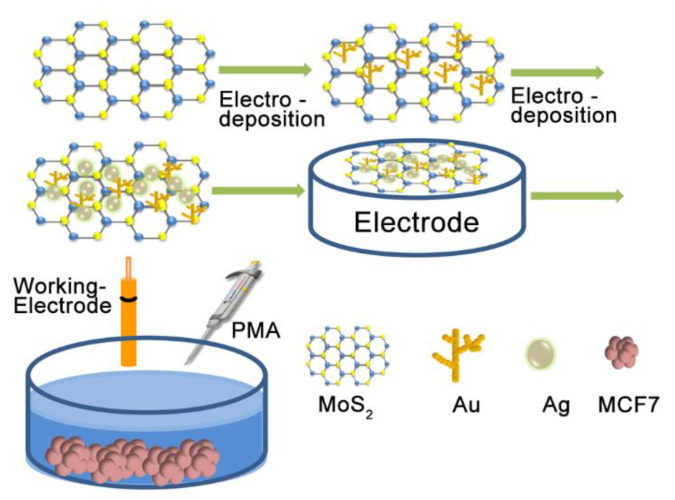
Fabrication and detection of the H_2_O_2_ electrochemical sensor.

The bare GCE was pre-polished with 0.3 µm and 0.05 µm alumina powders to get a smooth mirror surface before fabricating with a modified electrode [20]. For the purpose of wiping off the physically adsorbed substance, GCE was then sonicated with the mixture solution of ultra-pure water and ethanol for 10 min. After that, the cleaned electrode was dried under N_2_. Briefly, 10 µL of MoS_2_ suspension (2 mg mL^−1^) was cautiously dropped onto the GCE surface, and the modified electrode was dried under air. Then, MoS_2_/GCE was electrodeposited in 2.5 mM H[AuCl_4_] and 2.5 mM AgNO_3_ solutions, respectively, under the condition of −0.4 V. Accordingly, the MoS_2_-Au-Ag/GCE was successfully prepared. The whole process of the fabrication is illustrated in Scheme 1.

### 2.5. Detection of H_2_O_2_ Solutions with Different Concentrations and Released by Cells

The 30% pure H_2_O_2_ solution was diluted into 100 mM, 400 mM, 2000 mM and 4000 mM H_2_O_2_ with PBS, and the corresponding concentration of H_2_O_2_ was added to the 20 mL PBS buffer liquid system to conduct electrochemical detection of the modified electrodes. RPE-1 cells and MCF-7 cells were cultured in DMEM medium containing 10% fetal bovine serum (FBS), and were grown in a cell culture incubator with 5% CO_2_ and 37 °C. After they were grown to 80–90% confluence, the cells were washed 2–3 times with PBS, and resuspended into 2 mL oxygen-removing PBS for electrochemical detection of H_2_O_2_ generated by the MCF-7 cells. With the cell-free group as the control group and three groups of cells with different concentrations as the experimental group, the flux of H_2_O_2_ caused by normal cells and living cancer cells upon the addition of PMA at different concentrations and the correlated electrochemical response signals were recorded by amperometric i-t curves. The effect of the nanomaterials in the cell culture was assessed via an MTT assay and the detailed experimental steps can be found in Supporting Information.

## 3. Results and Discussion

### 3.1. Characterization of MoS_2_-Au-Ag/GCE Nanocomposite

**Figure 1:**
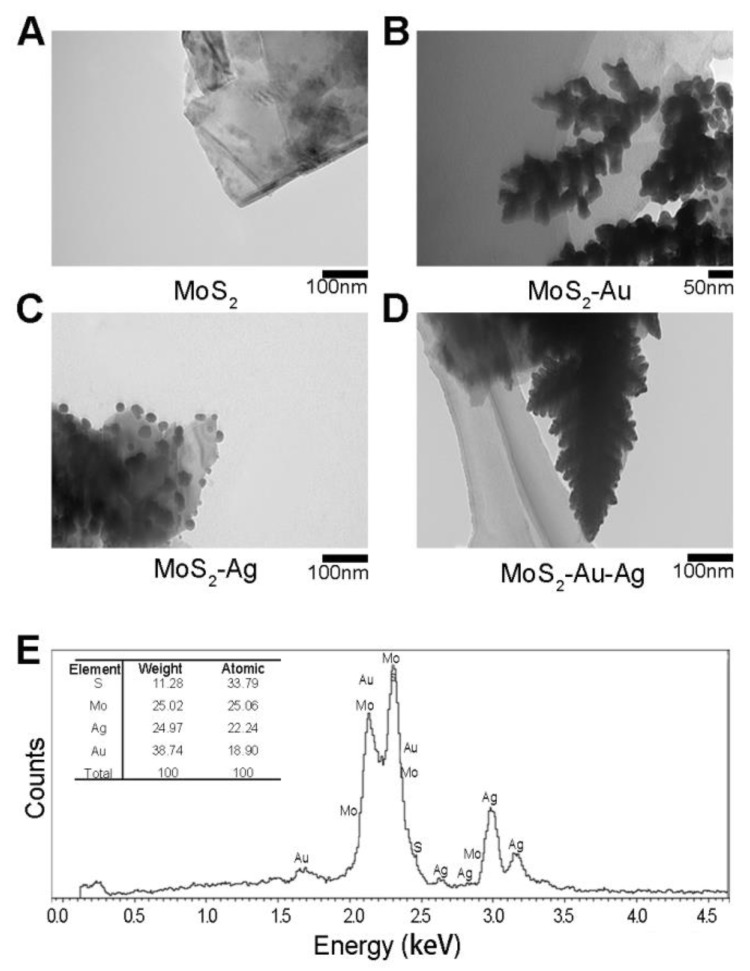
Characterization of the morphology, structure, and electrochemical performance of the nanocomposites. (**A**–**D**) Transmission electron microscopy images of MoS_2_ (**A**), MoS_2_-Au (**B**), MoS_2_-Ag (**C**) and MoS_2_-Au-Ag (**D**). (**E**) The X-ray energy dispersive spectrometric analysis of MoS_2_-Au-Ag.

**Figure 2:**
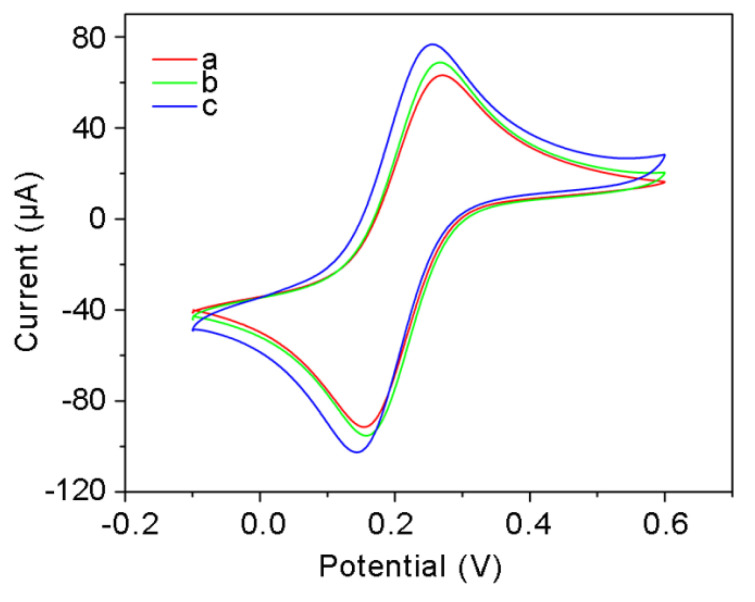
Cyclic voltammetry of nanomaterials. CV profiles of MoS_2_/GCE (a), MoS_2_-Au/GCE (b), MoS_2_-Au-Ag/GCE (c) recorded in K_3_[Fe(CN)_6_].

The morphology and structure of the obtained nanomaterials were investigated by TEM. Figure 1A shows that the MoS_2_ has a uniform lamellar structure. As illustrated in Figure 1B,C, the Au and Ag nanoparticles showed clear dendritic and granular morphologies, respectively, and were evenly dispersed over the MoS_2_. As shown in Figure 1D, the MoS_2_-Au-Ag mimics uniformly compact branches in the PDDA solution. In summary, we successfully synthesized the MoS_2_-Au-Ag nanocomposite, which has potential application value for improving electrochemical detection performance. The chemical composition of MoS_2_-Au-Ag was verified by XPS. As shown in Figure S1, the curve presented O 1s peaks, C 1s peaks, Ag 3d peaks, Mo 3d peaks, Au 4f peaks and S 2p peaks in one spectrum. For the Mo 3d region, the two characteristic peaks at 229.8 eV and 233.5 eV corresponded to Mo 3d_5/2_ and Mo 3d_3/2_ of MoS_2_, respectively, and the single S 2s peak is located at about 227 eV. Meanwhile, the binding energies at 162.1 eV and 163.2 eV can be attributed to the S 2p_3/2_ and S 2p_1/2_ species on the edges or surface of the MoS_2_ nanosheet, respectively. In addition, the strong peaks of the elements Au and Ag confirmed the successful electrodeposition of two metals on the glassy carbon electrode modified with MoS_2._ In addition, energy dispersive spectroscopy (EDS) was used to analyze and study the components of the MoS_2_-Au-Ag. The element of Mo, S, Au and Ag were clearly observed, which originated from the MoS_2_, Au and Ag nanomaterials, respectively (Figure 1E). The results further manifested that MoS_2_-Au-Ag was constructed successfully. We also studied the effects of different materials on sensor performance (Figure 2). The resulting spectra manifested that the oxidation and reduction peaks associated with MoS_2_-Au/GCE (b) and MoS_2_-Au-Ag/GCE (c) increased substantially compared with MoS_2_/GCE (a). Moreover, the peak value of redox was the largest for MoS_2_-Au-Ag/GCE, demonstrating that the Au-Ag nanocomposite particles have a good effect on enhancing electrode conductivity. The MoS_2_-Au-Ag composite was used to modify the electrode in the subsequent experiments.

### 3.2. Optimization of Experimental Parameters for the Fabrication of MoS_2_-Au-Ag/GCE

Changes in the experimental conditions of the modified electrode will directly affect the electrochemical properties of the electrode, which will lead to alterations of the catalytic properties and sensor functions [21]. For the sake of enhancing the sensing performance of the MoS_2_-Au-Ag/GCE, the effects of the PDDA concentration of dispersed MoS_2_, the electrodeposition time, and the modified electrode material were investigated.

**Figure 3:**
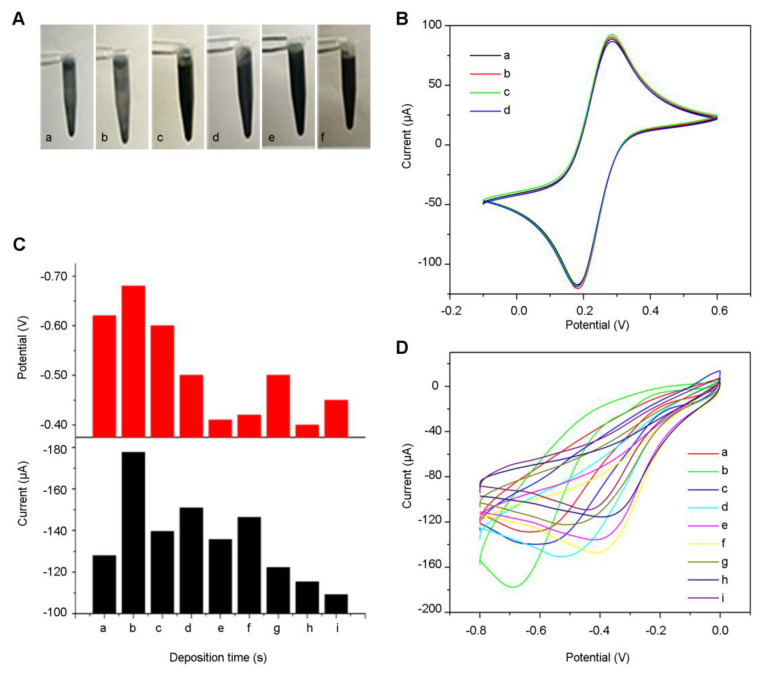
Optimization of experimental parameters for the fabrication of MoS_2_-Au-Ag/GCE. (**A**) MoS_2_ dispersion effects of 2 mg mL^−1^ water (a), 2 mg mL^−1^ PBS buffer (b), 5 mg mL^−1^ 0.5% PDDA (c), 5 mg mL^−1^ 1% PDDA (d), 2 mg mL^−1^ 0.5% PDDA (e) and 2 mg mL^−1^ 1% PDDA (f). (**B**) CV curves of dispersed MoS_2_ modified electrodes in K_3_[Fe(CN)_6_] under 2 mg mL^−1^ 0.5% PDDA (a), 2 mg mL^−1^ 1% PDDA (b), 5 mg mL^−1^ 1% PDDA (c), 5 mg mL^−1^ 0.5% PDDA (d). (**C**) The current line diagram of the electrode at 10 mM H_2_O_2_ with different deposition times. (**D**) CV curves of electrode modified with different depositions time in 10 mM H_2_O_2_. Deposition time: (a) Au 30 s Ag 30 s, (b) Au 30 s Ag 90 s, (c) Au 30 s Ag 150 s, (d) Au 90 s Ag 30 s, (e) Au 90 s Ag 90 s, (f) Au 90 s Ag150 s, (g) Au 150 s Ag 30 s, (h) Au 150 s Ag 90 s, (i) Au 150 s Ag 150 s.

MoS_2_ was dispersed with water, PBS buffer and PDDA of different concentrations, and then placed in a 4 °C refrigerator for 4 h after ultrasound to observe the dispersion (Figure 3A). The results showed that the MoS_2_ dispersed with water and PBS buffer had obvious precipitation and was not dissolved at all. The dispersion of MoS_2_ with PDDA was better, and a uniform MoS_2_ dispersion was obtained. The effect of PDDA concentration on the electrochemical property of MoS_2_-Au-Ag/GCE was analyzed by CV in K_3_[Fe(CN)_6_]. As shown in Figure 3B, the peak current values of 2 mg mL^−1^ MoS_2_ with 1% PDDA (b) and 5 mg mL^−1^ MoS_2_ with 1% PDDA (c) are similar. Based on the principle of economy, 2 mg mL^−1^ MoS_2_ with 1% PDDA was selected for subsequent experiments. The Au-Ag electrodeposited on the surface of MoS_2_/GCE can provide a large effective surface area and enhance the electrocatalytic performance of the electrode, which is of great significance for the determination of H_2_O_2_ [22]. Thus, the amount of Au-Ag electrodeposited on the surface of MoS_2_/GCE immediately affects the performance of the H_2_O_2_ sensor. To enhance the function of Au-Ag on the MoS_2_/GCE, the effect of electrodeposition time from 30 s to 150 s was analyzed by CV in 10 mM H_2_O_2_, as shown in Figure 3D. The analysis results revealed that the potential and the current value of redox peak were different. As we can see from Figure 3C, the b and d peak current values are bigger, but the peak potential value of b is larger. As such, we chose the optimized electrodeposition time in the fabrication of modified electrode to reduce the interference of high voltage.

### 3.3. Electrocatalytic Activities of the Modified Electrodes

**Figure 4:**
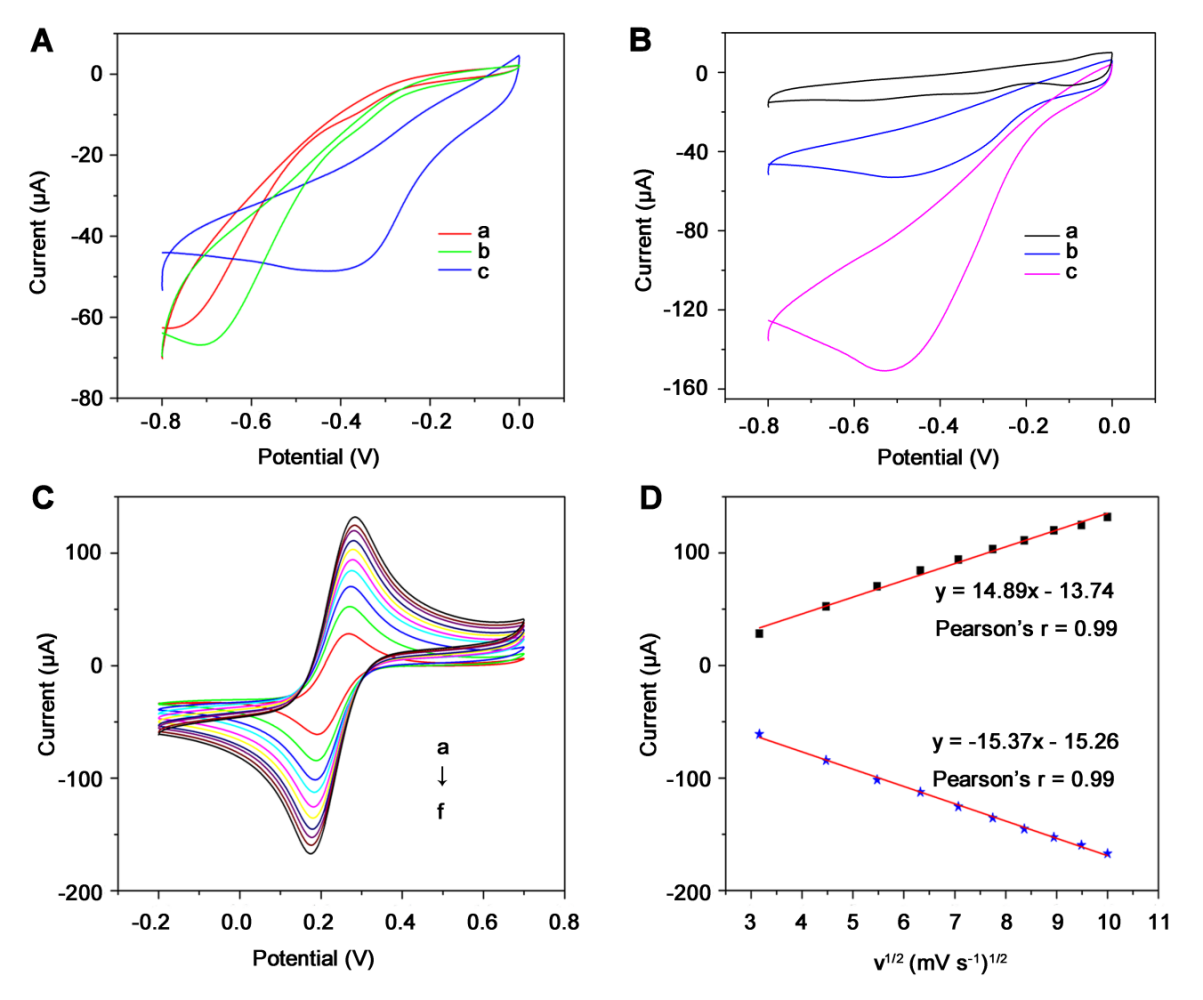
Electrocatalytic activities of the modified electrodes. (**A**) CV profiles of electrode modified by MoS_2_/GCE (a), MoS_2_-Au/GCE (b) and MoS_2_-Au-Ag/GCE (c) in 5 mM H_2_O_2_. (**B**) CV curves of MoS_2_-Au-Ag/GCE in PBS (a), 5 mM H_2_O_2_ (b) and 10 mM H_2_O_2_ (c). (**C**) Kinetic analysis of MoS_2_-Au-Ag/GCE at scan rates ranging from 10 to 100 mV s^−1^. (**D**) Linear fits of the oxidized peak current (*I_pa_*) and reduced peak current (*I_pc_*_)_ versus the square root of the scan rate (v^1/2^).

We employed CV to research the electrocatalytic activities of MoS_2_/GCE, MoS_2_-Au/GCE and MoS_2_-Au-Ag/GCE in 5 mM H_2_O_2_ (Figure 4A). The CV experiments revealed that no redox peaks were measured relating to MoS_2_/GCE (a) in the range of −0.80 V–0.00 V, while MoS_2_-Au/GCE (c) and MoS_2_-Au-Ag/GCE (d) had the presence of a reduction peak of H_2_O_2_ at −0.50 V. Therefore, MoS_2_-Au-Ag/GCE had a better reaction performance to H_2_O_2_.

We then examined the CV results of MoS_2_-Au-Ag/GCE in PBS and in different concentrations of H_2_O_2_ (Figure 4B). The redox peak of modified electrodes was not measured in PBS (a) while it was shown in 5 mM (b) and 10 mM H_2_O_2_ (c). With the increase in concentration of H_2_O_2_, the reductive peak position shifting to the left may be due to the delay of electron transfer caused by the increase in H_2_O_2_ concentration. In order to prevent interference from other substances, −0.45 V was selected as the catalytical voltage for subsequent current response experiments. Compared with the 5 mM H_2_O_2_ solution, the reduction peak current value measured by the modified electrode in 10 mM H_2_O_2_ increased significantly, indicating that MoS_2_-Au-Ag/GCE had a higher sensitivity to H_2_O_2_.

The kinetics of the MoS_2_-Au-Ag/GCE were investigated by analyzing the impacts of the scan rate on the redox current. The electrochemical performance of MoS_2_-Au-Ag/GCE was examined in 10 mM K_3_[Fe(CN)_6_] with the increase of scan rate from 10 to 100 mV s^−1^. As displayed in Figure 4C, a great linear relationship was shown between the square root of scan rate and the peak current density. The maximum current of the redox peak was increased linearly and the distance of the peak was expanded gradually. According to these results, we made a linear fit of the oxidation peak (*I_pa_*) and reduction (*I_pc_*) peak currents with the square root of the scan rate (v ^1/2^). The relevant linear regression equations were *I_pa_* = 14.89 v^1/2^ − 13.74 and *I_pc_* = −15.37 v^1/2^ − 15.26 with correlation coefficients of 0.9916 and 0.9976, respectively (Figure 4D). The electrochemical signal of the modified electrode suggests that the catalysis process is diffusion controlled.

### 3.4. Amperometric Responses to H_2_O_2_

**Figure 5:**
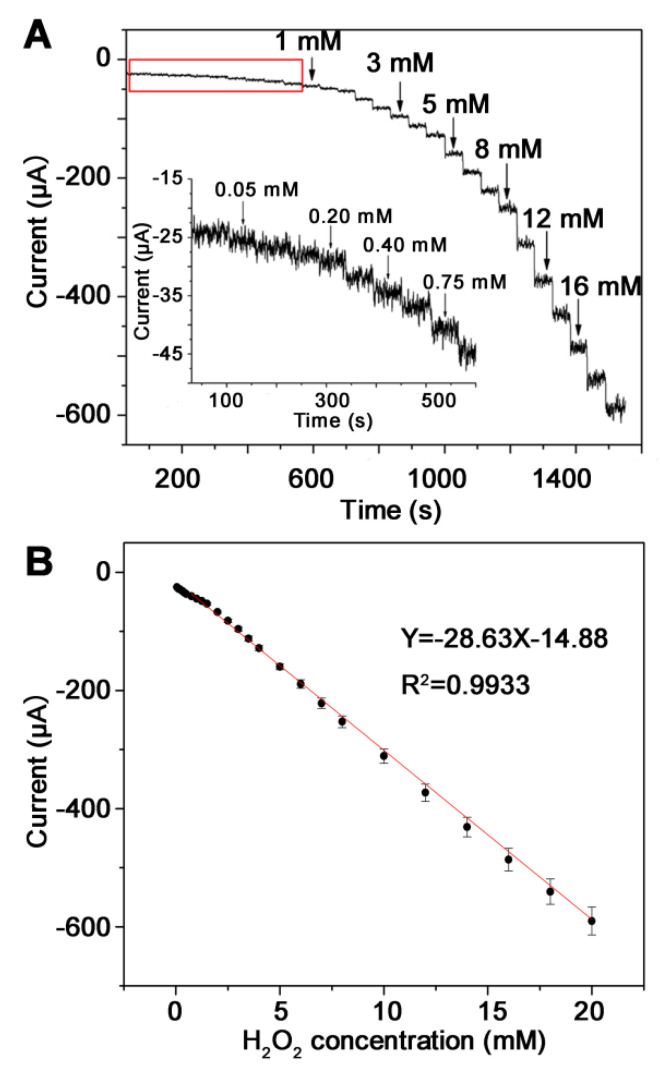
Kinetics of MoS_2_-Au-Ag/GCE. (**A**) The i-t curve of the MoS_2_-Au-Ag/GCE when different concentrations of H_2_O_2_ were added to the PBS buffer successively. (**B**) Linear fitting diagram.

To further detect the amperometric current responses of MoS_2_-Au-Ag/GCE, we added H_2_O_2_ at different concentrations continuously at a potential of −0.45 V. To prevent interference, H_2_O_2_ was added for the first time after the current was kept at a stable value, and then added every 50 s. Figure 5A shows the amperometric response of MoS_2_-Au-Ag/GCE with various concentrations of H_2_O_2_. After injecting a certain amount of H_2_O_2_, MoS_2_-Au-Ag/GCE exhibited a fast current response because of the excellent electrocatalytic performance of MoS_2_-Au-Ag. It can be clearly seen that each reduction current response demonstrated an explicit increase as the concentration of H_2_O_2_ increased and the response current attained a steady state within 4 s. The illustration manifests the amperometric current response at lower concentrations of H_2_O_2_ (0.05 mM–0.75 mM).

As shown in Figure 5B, the calibration curve was fitted by using multiple sets of repeated experimental data. According to the curve fitting, we calculated the linear range of the prepared sensor to be from 0.05 mM to 20 mM, with a linear correlation coefficient of 0.9933, and the sensitivity was 405.24 µA mM^−1^ cm^−2^ (28.63 µA mM^−1^). It is worth noting that the sensitivity is much higher than many reported H_2_O_2_ sensors, as shown in Table 1. The detection equation of the sensor was Y = −28.63 X − 14.88 and the minimum detection limit was 7.19 µM. This experiment further demonstrated that the MoS_2_-Au-Ag composite is an excellent material for H_2_O_2_ detection with good sensitivity.

### 3.5. Repeatability and Stability of MoS_2_-Au-Ag/GCE

**Figure 6:**
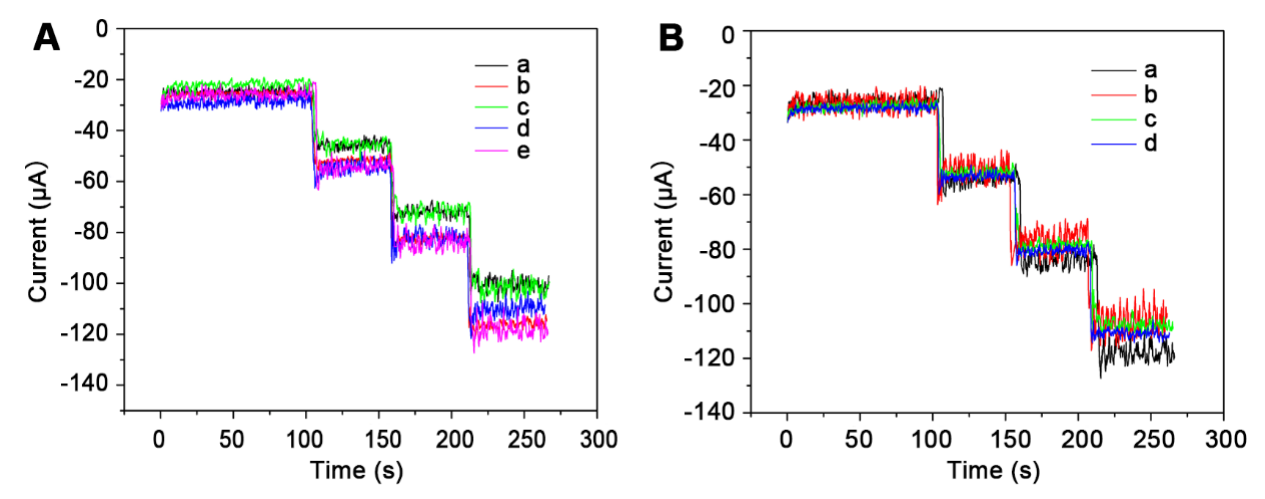
Results obtained using the H_2_O_2_ sensor are highly sensitive and reproducible. (**A**) The i-t curves of MoS_2_-Au-Ag/GCE were dripped with the same concentration of H_2_O_2_ into PBS. (**B**) The i-t curves of the same MoS_2_-Au-Ag/GCE when the same concentration of H_2_O_2_ was dripped into PBS after the electrode was placed at different times.

The repeatability and stability of the electrochemical sensor are the main parameters affecting the applicability of the H_2_O_2_ sensor. The current response values of five modified electrodes when 1 mM H_2_O_2_ was successively injected under the same conditions are recorded in Figure 6A. The RSD of the relative standard deviation of the five electrodes was calculated to be 3.79%. The stability was evaluated by placing the MoS_2_-Au-Ag/GCE in PBS buffer and adding 1 mM H_2_O_2_ continuously (Figure 6B). The modified electrode was tested every 4 h and then placed overnight in a 4 °C refrigerator for further testing. The response current of the i-t curve detected for the first time at a large concentration has some deviation from other curves, but the deviation is small, and the other curves almost coincide, yielding an RSD of 3.09%. Thus, the MoS_2_-Au-Ag electrode shows a good repeatability and an acceptable stability, indicating that the MoS_2_-Au-Ag composite material has a good application prospect in H_2_O_2_ detection.

### 3.6. Detection of H_2_O_2_ Released from Normal Cells and Living Cancer Cells

**Figure 7:**
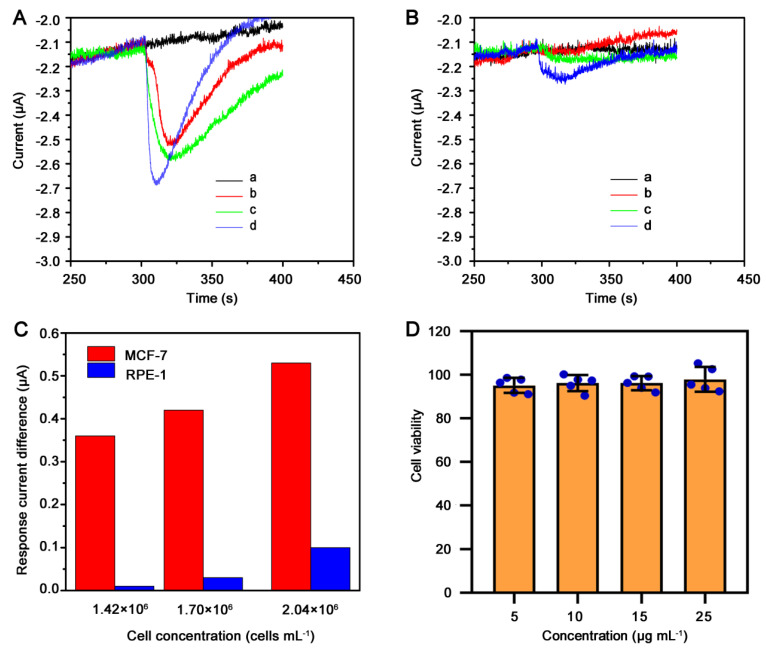
Detection of H_2_O_2_ released from normal cells and living cancer cells. (**A**) Amperometric response of MoS_2_-Au-Ag/GCE to injection of 200 ng mL^−1^ PMA with MCF-7 cells in 0.1 M PBS (pH 7.4). (**B**) Amperometric response of MoS_2_-Au-Ag/GCE to injection of 200 ng mL^−1^ PMA with RPE-1 cells in 0.1 M PBS (pH 7.4). (**C**) Comparison of response current differences between MCF-7 and RPE-1 cells induced by H_2_O_2_ release. (**D**) Effects of prepared MoS_2_-Au-Ag on metabolic activities of MCF-7 cells.

H_2_O_2_ is an important representative of the reactive oxygen species related to lots of cellular life activities, and is a potential biomarker for cancer cells [28,29,30]. In order to better explore the physiological processes of early-stage cancer cells, the monitoring of H_2_O_2_ levels in the cell environment is crucial. In this study, we selected human breast cancer cells (MCF-7) for the on-site real-time monitoring of H_2_O_2_ release after stimulation. First, an MTT assay was used to evaluate the effects of the prepared nanomaterials on living cancer cells. The results showed that the survival rate of these living tumor cells all reached more than 90% after 24 h of co-incubation, even with 25 μg mL^−1^ nanomaterials, so the cell growth was almost unchanged and the original activity was maintained (Figure 7D). Therefore, it can be proven that the MoS_2_-Au-Ag nanocomposite was biocompatible with living cells and can be used for further electrochemical tests. Moreover, the dynamic release of H_2_O_2_ by pure a PBS solution and MCF-7 cells of different concentrations was detected using the MoS_2_-Au-Ag sensor before and after the stimulation of PMA. As depicted in Figure 7A, we observed a significantly reduced current increase upon the addition of 40 µL PMA (0.5 mg mL ^−1^) when the PBS was suspended with MCF-7 cells. The control group with the absence of MCF-7 cells (a) showed no detectable current response to the injection of PMA, indicating that the triggered H_2_O_2_ was the result of PMA-stimulated cells. In addition, curves b, c, and d showed the current response curve of the MoS_2_-Au-Ag sensor at –0.4 V in the presence of the 10%, 30% and 50% concentrations of MCF-7 cells by physiological injection of PMA, respectively. The results revealed that the reduction current increases more significantly as the cell concentration increases. Besides, hydrogen peroxide, released by human retinal pigment epithelial cells (RPE-1), was detected under the same conditions. As shown in Figure 7B, we observed that the control group (a) without RPE-1 cells showed no current response after PMA injection, while the experimental group with PMA stimulation showed a slight decrease in current. Compared with the MCF-7 cells, RPE-1 cells showed a very weak reduction in response current, further indicating that the triggered H_2_O_2_ was the result of the stimulation of cancer cells by PMA, as exhibited in Figure 7C. In conclusion, the proposed MoS_2_-Au-Ag sensor could be utilized to detect the H_2_O_2_ released by living cancer cells, and it has the potential to be used as a physiological and pathological H_2_O_2_ sensor for the diagnosis and treatment of cancer in its early stages.

## 4. Conclusions

In summary, we constructed an H_2_O_2_ sensor based on MoS_2_-Au-Ag nanocomposites by electrodepositing Au and Ag nanoparticles to modify MoS_2_/GCE. The resulting MoS_2_-Au-Ag/GCE exhibited an excellent electrochemical performance with a sensitivity of 405.24 µA mM^−1^ cm^−2^ (28.63 µA mM^−1^) and a linear detection range of 0.05 mM to 20 mM. The H_2_O_2_ sensor has good repeatability and stability, and a short response time. The H_2_O_2_ sensor constructed in this study has also been successfully applied in the detection of H_2_O_2_ generated by living cancer cells, indicating that this work is expected to play a significant role in the early diagnosis and treatment of tumors and providing an important basis for the development of effective treatment strategies. Thus, we believe that the prepared MoS_2_-Au-Ag sensor can be used to analyze H_2_O_2_ in all sorts of samples, and we can continue to miniaturize it for better clinical testing in the future.

## Figures and Tables

**Table 1:** Comparison of the performances of various H_2_O_2_ sensors.

Material	Sensitivity (µA mM^−1^ cm^−2^)	Detection Limit (µM)	Linear Range (mM)	Reference
Pt/OMCs	184.6	1.2	0.5–4.5	[23]
Ag-TiO_2_	65.23	0.83	0.0083–0.0433	[24]
ZIF-67/CNFs	323	0.62	0.0025–0.19	[25]
Pt-SnO_2_@C	241.1	0.1	0.001–0.17	[26]
PANI/HRP/SWCNTs	200	900	2.5–50	[27]
MoS_2_-Au-Ag	405.24	7.19	0.05–20	this work

## Supplementary Materials

The following are available online at https://www.mdpi.com/1424-8220/20/23/6817/s1, Figure S1: XPS survey spectrum of MoS_2_-Au-Ag.
