# Supplementary material for: An Electrochemical Sensor Based on Chalcogenide Molybdenum Disulfide-Gold-Silver Nanocomposite for Detection of Hydrogen Peroxide Released by Cancer Cells
Source: Sensors (Basel). 2020 Nov 28;20(23):6817. doi: 10.3390/s20236817 (PMC7730666; doi:10.3390/s20236817)
Supplement: Supplementary file 1 [file sensors-20-06817-s001.pdf]

Supporting Information

An electrochemical sensor based on chalcogenide molybdenum disulfide-gold-silver nanocomposite for detection of hydrogen peroxide released by cancer cells

Jinchun Hu[+], Congcong Zhang[+], Xue Li , Xin Du*

Shandong Provincial Key Laboratory of Animal Resistance Biology, Key Laboratory of Food Nutrition and Safety, College of Life Sciences, Shandong Normal University, Jinan, Shandong 250014, China.

*Corresponding author

E-mail address: xdu@sdnu.edu.cn (X. Du)

1. EXPERIMENTAL SECTION

1.1 MTT assay

MCF-7 cells (human breast cancer cells) were purchased from the cell bank of the Chinese Academy of Sciences and cultured in the laboratory of Shandong Normal University. The standard MTT method was employed to study the biological activity of cells. The process of the cytotoxicity assay by MTT method is as follows: The 96-well cell culture plate was inoculated with 5×10^3 MCF-7 cells per well and incubated at 37 °C under 5% CO_2 for 24 hours. Then, we aspirated the old cell culture medium and added 100 μL of new culture medium containing different concentrations of the proposed MoS_2-Au-Ag nanocomposites to incubate for 24 hours. The group without nanoparticles was used as the control group, and the group with the concentration gradient of nanocomposites of 5 μg mL^{-1}, 10 μg mL^{-1}, 15 μg mL^{-1}, 25 μg mL^{-1} was used as the experimental group. After the cells were washed twice with PBS, 20 μL of the MTT solution (5 mg mL^{-1} in PBS, pH 7.4) was added to each well and incubated for 4 h at 37 °C. The supernatant was removed from each well by using a pipette, and the products were dissolved with dimethyl sulfoxide (100 μL). Subsequently, we placed the 96-well plate on a shaker and shook for 10 minutes, and then used a microplate reader to detect the absorbance of the cells in each well at the wavelength of 570 nm. 5 replicate wells were designed in each group of cell experiments, and the relative cell viability was calculated based on the ratio of the absorbance of the experimental group to the untreated control cells.

2. PICTURE SECTION

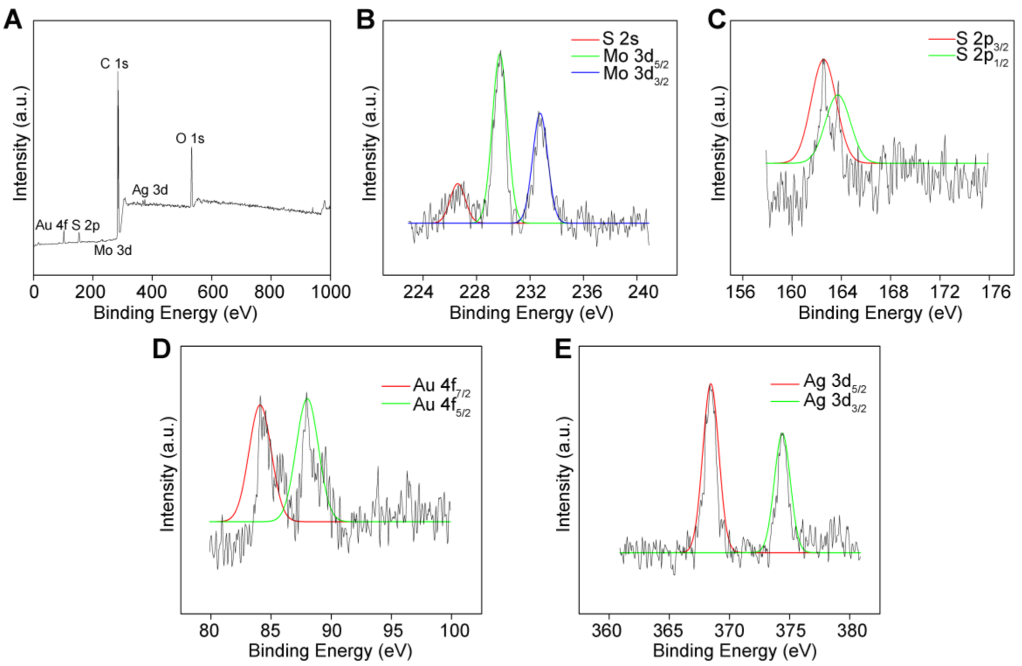

Figure S1. XPS survey spectrum of MoS_2-Au-Ag (A), and XPS high-resolution spectra of MoS_2-Au-Ag composites: Mo (B), S (C), Au (D) and Ag (E).
